# Stakeholder Perspectives on Barriers and Facilitators on the Implementation of the 1000 Days Plus Nutrition Policy Activities in Ghana

**DOI:** 10.3390/ijerph18105317

**Published:** 2021-05-17

**Authors:** Anne Galaurchi, Samuel T. Chatio, Paula Beeri, Abraham R. Oduro, Winfred Ofosu, Mark Hanson, Marie-Louise Newell, Shane A. Norris, Kate A. Ward, Engelbert A. Nonterah, Regien Biesma, on behalf of the INPreP Study Group

**Affiliations:** 1 Global Health Unit, Department of Health Sciences, University Medical Center Groningen (UMCG), 9700 RB Groningen, The Netherlands; r.biesma@umcg.nl; 2 Navrongo Health Research Centre, Ghana Health Service, Navrongo 00233, Ghana; schatio@yahoo.co.uk (S.T.C.); Paula.beeri@navrongo-hrc.org (P.B.); aroduro@gmail.com (A.R.O.); drenanonterah@gmail.com (E.A.N.); 3 Upper East Regional Health Directorate, Ghana Health Service, PMB, Bolgatanga 00233, Ghana; winfred.ofosu@ghsmail.org; 4 Institute of Developmental Sciences and NIHR Biomedical Research Centre, University of Southampton and University Hospital Southampton, Southampton SO17 1 BJ, UK; M.Hanson@soton.ac.uk; 5 Department of Human Development and Health, Faculty of Medicine, University of Southampton, Southampton SO17 1 BJ, UK; M.Newell@soton.ac.uk; 6 SAMRC Developmental Pathways for Health Research Unit (DPHRU), Department of Paediatrics, Faculty of Health Sciences, University of the Witwatersrand, Johannesburg 2193, South Africa; Shane.Norris@wits.ac.za (S.A.N.); kw@mrc.soton.ac.uk (K.A.W.); 7 Global Health Research Institute, School of Health and Human Development, University of Southampton, Southampton SO17 1 BJ, UK; 8 Medical Research Council Lifecourse Epidemiology Unit, University of Southampton, Southampton SO17 1 BJ, UK; 9 Julius Global Health, Julius Center for Health Sciences and Primary Care, University Medical Center Utrecht, Utrecht University, 3508 GA Utrecht, The Netherlands; 10 INPreP Study Group

**Keywords:** maternal child health, nutrition, first 1000 days, 1000 days plus, qualitative research, Ghana, Sub-Saharan Africa, health policy implementation, barriers, stakeholder analysis

## Abstract

Optimizing nutrition in the preconception and 1000 days periods have long-term benefits such as higher economic productivity, reduced risk of related non-communicable diseases and increased health and well-being. Despite Ghana’s recent progress in reducing malnutrition, the situation is far from optimal. This qualitative study analyzed the maternal and child health nutrition policy framework in Ghana to identify the current barriers and facilitators to the implementation of nutrition policies and programs relating to the first 1000 days plus. Data analyzed included in-depth interviews and focus group discussions conducted in Ghana between March and April 2019. Participants were composed of experts from government agencies, civil society organizations, community-based organizations and international partners at national and subnational levels. Seven critical areas were identified: planning policy implementation, resources, leadership and stakeholders’ engagement, implementation guidance and ongoing communication, organizational culture, accountability and governance and coverage. The study showed that, to eradicate malnutrition in Ghana, priorities of individual stakeholders have to be merged and aligned into a single 1000 days plus nutrition policy framework. Furthermore, this study may support stakeholders in implementing successfully the 1000 days plus nutrition policy activities in Ghana.

## 1. Introduction

**Figure 1:**
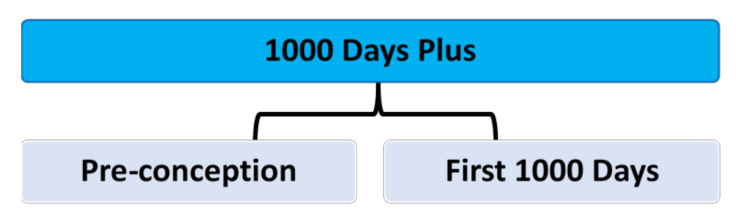
The 1000 days plus concept: pre-conception phase and first 1000 days (pregnancy and first two years of infancy) [4].

Malnutrition is a major global health challenge. Women, infants, children and adolescents are especially at risk [1,2]. In 2008, for the first time, the Lancet series on Maternal and Child Undernutrition acknowledged the “first 1000 days” as the most crucial period to address malnutrition [3]. This vulnerable period starts from conception until the age of two years. Research has shown that optimizing nutrition in the first 1000 days ensures the best possible start in life, setting the basis for lifelong health and well-being [4,5,6]. In particular, Developmental Origins of Health and Disease (DOHaD) research demonstrated how early life under-nutrition coupled with excessive weight gain in later life significantly increases risk for adult obesity, diabetes and hypertension [7]. In 2016, the Lancet commission on adolescent health and well-being indicated that, in addition to the first 1000 days, the adolescent period plays a crucial role in malnutrition [8]. Adolescence, considered as the pre- and early parenthood phase, has the potential to produce a “triple dividend” benefiting young parents now, their future adult life and the lives of their children [8,9]. In the same year, the United Nations Global strategy for Women’s, Children’s and Adolescents’ health recognized the preconception period as a window of opportunity to tackle malnutrition [10]. In response to this, the “1000 days plus” concept was created by uniting the pre-conception phase to the first 1000 days (see Figure 1). Studies show that countries investing in this concept have higher economic productivity and promote health and well-being by decreasing the risk of related non communicable diseases (NCDs) [11,12,13,14].

In the past decade, Ghana has made progress in improving nutrition status through the implementation of some 1000 days plus nutrition policies. These policies proved effective even though they were targeting under- and overnutrition separately instead of addressing it as “double-duty interventions” for all forms of malnutrition, which has recently been proposed as a more holistic approach [15,16,17]. In fact, between 2008 and 2014, stunting rates in Ghana fell from 28% to 19% [6]. In 2011, Ghana became part of the Scaling Up Nutrition (SUN) movement. This led to the formation of the National Nutrition Partner Coordination (NaNuPac), part of the Cross Sectoral Planning Group (CSPG) platform, to support policy implementation aspects of nutrition service delivery [5,18]. In the same year, Ghana teamed up with the Renewed Efforts Against Child Hunger (REACH) initiative [19]. In 2012, Ghana committed to the global targets of improving maternal, infant and young child nutrition by 2025 [20]. These targets were then eventually translated and incorporated into the National Nutritional Policy (NNP) in 2013 [19]. The NNP is a collection of different individual policies and programs that jointly contribute to a broader policy framework for nutrition and which includes, but is not limited to, the 1000 days plus nutrition period. In the NNP, the 1000 days plus nutrition policy activities include both nutrition-sensitive and nutrition-specific policies [3]. Nutrition-sensitive policies and programs address the broader determinants of malnutrition and focus on improving health and other sectors such as education, water and sanitation and food and agriculture, as well as and gender and social protection [5,20,21]. Nutrition-specific policies and programs focus on the immediate determinants of child nutrition, such as growth promotion, micronutrient supplementation and health promotion programs. In Ghana, these policies are delivered by the Nutrition Department which is a sub-unit of the Ghana Health Service (GHS). However, the Nutrition Department is inadequately resourced to carry on its mandate [5,21].

Despite Ghana’s recent progress in reducing malnutrition, micronutrient deficiencies among women and children remain a major public health challenge [22]. Ghana is now facing a “double burden” of malnutrition. Undernutrition in childhood is followed by overweight with micronutrient deficiencies prevalent throughout infancy and childhood [23]. The 2014 Ghana Demographic and Health Survey showed that about 20% of children under five years are stunted. Similarly, micronutrient deficiency (especially vitamin A, iodine and iron) is common among women and children. Among children under five years, 66% were anemic, while 44% of women 15–49 years were anemic [23]. The survey also indicated that about 40% of women 15–49 years in Ghana were obese and that obesity (especially among women) is steadily growing in the Ghanaian society. The prevalence of the different types of malnutrition greatly varies between regions. The rates of stunting and anemia in the Northern and Upper East regions remain above the national average [24]. As the government also acknowledged, the implementation of the nutrition policies and programs in the 1000 days plus policy framework presents several challenges [21].

In low- and middle-income countries (LMIC) such as Ghana, where resources are scarce, investing in services that produce the highest health gain “return on investment” is critical [25]. The 1000 days plus activities and programs have the potential to achieve societal and economic gains in the long term [3,8,9,10]. Nevertheless, policies alone are not sufficient. Without effective implementation, even high-quality policies will lead to failure. Therefore, it is important to identify possible challenges during the implementation phase and reduce the implementation gap between the policy goals and the outcome [26]. In the case of Ghana, the implementation status of nutrition policies and programs in the 1000 days plus period is unknown. Policy implementation monitoring and evaluation, key processes used for determining whether the goals are being reached, are not fully in place. Therefore, there is a need to evaluate the current implementation of the 1000 days plus activities to guide decision-makers. This study was developed in a specific effort to be useful and applicable to the work of GHS and other stakeholders who are engaged in the implementation of the nutrition policies in the 1000 days plus period. This study aims to evaluate the current policy implementation process of the 1000 days plus initiatives in Ghana. In particular, in this qualitative research we sought to identify the prevailing main barriers and facilitators to the implementation of nutrition policies within the 1000 days plus policy landscape in Ghana. The results may guide the implementation and support the development of appropriate policies and programs aimed at improving nutrition in the first 1000 days plus in Ghana.

## 2. Materials and Methods

This is a sub-study of a larger UK NIHR-funded international collaborative study, the Improved Nutrition Preconception Pregnancy Post-Delivery (INPreP), based in Ghana, Burkina Faso, South Africa and Southampton, UK. INPreP aims to develop supportive delivery of double duty nutrition interventions in the 1000 days plus [15,16]. To address the objectives of the INPreP study, six work packages were formulated. This current study is part of work package one (WP1) also called *Policy Landscape.* WP1 sought to understand the policy landscape regarding maternal and child health and nutrition before, during and after pregnancy [13]. It involves the review of policy documents, online and hard copies and stakeholder engagement of experts in the field. The first part of WP1 was a policy review which aimed to summarize the national focus regarding 1000 days plus and nutrition policy, within the framework of Maternal and Child Health. The second part was a stakeholder engagement study which aimed to evaluate the current policy implementation process of the 1000 days plus policy framework.

These analyses are based on WP1 data collected in Ghana between March and April 2019. Qualitative data were collected from in-depth interviews (IDIs) conducted between March and April 2019 and stakeholder panel discussions, in the form of focus group discussions (FGDs) held on 10 April 2019 in Navrongo, Ghana. Both IDIs and FGDs were employed to obtain an integral understanding of the nutrition policies and programs in the 1000 days plus period in Ghana. The IDIs provided an opportunity to examine the individual experiences and to compare differences and similarities among stakeholders, whereas the FGDs, based on the interaction and combination of the experiences of multiple stakeholders, provided a deeper understanding of specific or priority research areas.

The study received ethics approval from the Navrongo Health Research Centre’s Institutional Review Board (Approval number: NHRCIRB325). Informed consent was obtained from all participants prior to both IDIs and FGDs.

### 2.1. Setting

The research was carried by the Navrongo Health Research Centre (NHRC) located in the northern part of Ghana. NHRC runs a Health and Socio-Demographic Surveillance System in the Kassena-Nankana districts [27]. Ghana is a middle-income country located in the West African coast and has a GDP of 66.98 billion USD (2019) with an annual growth of 6.5% [28]. It is one of the countries with a high burden of malnutrition with stunting rates estimated to be 20% in children less than five years of age. Healthcare delivery in the country comprises public which is mainly governmental, private and traditional medicine administered by herbal and faith-based organizations. Most healthcare services are provided by the government and administered by the Ghana Health Services (GHS). The government healthcare system is divided into five levels of service provision: community-based health planning and services (CHPS) compounds, health centers and polyclinics and district, regional and tertiary hospitals (teaching hospitals). Health programs are often funded by the government, the national health insurance scheme, internally generated funds (IGF) and pooled donors’ health funds. Similarly, healthcare policies are often initiated by the Ghana Health Service and Ministry of Health with the support of donors. Certain healthcare policies are often initiated by donors and implemented by the GHS giving a “top-down” approach to policy implementation [27]. The current study therefore utilized stakeholders who work within various sectors and departments of the health system structure to enable complete saturation of the participants.

### 2.2. Sampling Strategy

This study employed purposive sampling with snowballing until saturation was reached. Purposive sampling is a technique widely used in qualitative research to identify and select individuals (or groups) who are especially knowledgeable about a phenomenon of interest [18,29,30]. For individual semi-structured interviews, recruitment started with the research team drafting a list of relevant multi-sector, multi-actor and multi-level stakeholders involved in the broad areas of maternal and child health and nutrition. Representatives at the local, national and sub-national level from government, civic society and United Nations (UN) agencies, from healthcare to social welfare, education and agriculture, were identified. The stakeholders were contacted and invited to participate in the in-depth interviews. From the first group of respondents, using the snowball technique, more research participants were then selected and recruited according to their relevance to the researched topic. After the IDIs, the participants were invited to join the upcoming stakeholder panel discussions. These discussions in the form of FGDs brought together the stakeholders who were interviewed for the IDIs to a common venue for joint discussions. Two separate FGDs, with a total of 30 participants, were conducted. Since FGDs involve on-the-spot interaction, and are therefore more difficult to moderate, a maximum number of 15 stakeholders per FGD was established. The research team divided the participants into two homogeneous groups based on the sector, operational level and organization of the participants. At the end of the study, 11 in-depth interviews and 2 parallel FGDs were conducted. In particular, all the operational levels of the healthcare system at community, district, regional and national level were represented as well as multinational organizations. Based on the main criteria in assessing sampling saturation, we concluded that the researched population was a good reflection of the study population (see Table 1).

### 2.3. Data Collection

The primary data were collected through IDIs and FGDs with experts. The main aim was to explore the national nutrition, maternal and child health policy landscape with a focus on policies related to optimizing the nutritional status in the 1000 days plus. There were six areas of interest that formed the basis of the topic guide during the interviews: current activities, priorities, best entry points, key players, challenges and research support. During both the IDIs and FGDs, each research participant was questioned based on these criteria. The 11 IDIs were conducted alternatively by two experienced qualitative researchers from the Navrongo Health Research Centre at the workplace or office of the research participants. A semi-structured topic guide, composed mainly of open questions, was used to assist researchers in covering the main areas of interest with flexibility to probe further for vital information. Written consent was obtained prior to each interview. The interviews, which lasted from 15 to 25 min, were conducted in English, audio recorded and transcribed. The FGDs took place at the conference hall at the Navrongo Health Research Centre. The agenda for the day was organized into two sections; first was a series of three sequential presentations followed by breakout sessions for the actual FGD. On arrival, each participant signed an attendance book which also served as additional consent for participation in the FGDs. The presentations started with a brief introduction to the study team and the expert stakeholders. After the presentations, the participants were then divided into two groups for the FGDs. The two FGDs took place in parallel in different rooms within the conference hall. For each FGD, there were two experienced qualitative researchers from the Navrongo Health Research Centre acting as a moderator and note-taker. Inclusiveness played a crucial role in the setting. The moderator had the skills to include each participant in the conversation and ensure that for every discussion point different perspectives were presented. In addition to the moderator and note taker, the study coordinator and principal investigator were also present to ensure that all main areas of interest were addressed. A topic guide was used to help ensure that areas of prior interest to the research team were explored in each FGD. The FGDs were audio recorded and subsequently transcribed.

### 2.4. Data Analysis

Audio recordings from the IDIs and FGDs were transcribed verbatim by members of the research team (mainly research assistants with first degree qualification or research officers with a master’s degree). The in-depth interviews were individually transcribed, whereas the FGDs were double transcribed. Contrary to the IDIs, the FGDs were transcribed verbatim by one researcher and eventually quality controlled by another researcher to ensure the accuracy of the transcript. Directed qualitative content analysis, also known as thematic or framework analysis, was used to analyze the data. Data management and coding was performed using Atlas Ti software (ATLAS-LAS.ti Scientific Software Development GmbH, Berlin, Germany). The transcribed interviews were coded to identify key themes and establish patterns. A six-phased approach was used for conducting the data analysis [30]. The first phase included initial familiarization with the data followed by the generation of initial codes. Consequently, the transcripts were systematically coded according to initial codes and the data were then scanned to identify potential emerging themes. Themes were then selected, named and defined.

## 3. Results

**Figure 2:**
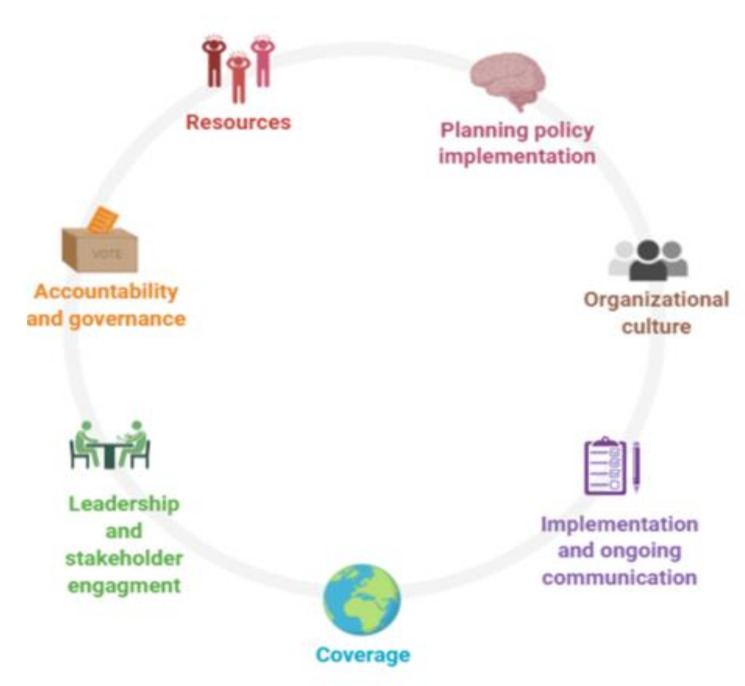
Seven critical areas for action in the implementation of the 1000 days plus activities in Ghana.

From the analysis of the in-depth interviews with key stakeholders and expert panel discussions, seven core themes (as illustrated in Figure 2) and ten sub-categories were identified. These seven themes are the result of different coding rounds that emerged from the six-phased thematic analysis. In general, the codes that reached the highest score were: “funds”, “monitoring system” and “capacity building”.

### 3.1. Planning Policy Implementation

#### 3.1.1. Define Problem and Set Goals

There was a general agreement among participants that insufficient planning for 1000 days plus policies prevented its full implementation. Often this started with a lack of problem definition and its related policy goals. Government and civil society representatives reported that they mainly set their priorities based on donor agencies’ criteria rather that their own problem analysis. This enabled them to receive donors’ funds. Even when the problem was identified by stakeholders at the local level, it was difficult to advocate it to the policymakers, as an NGO representative mentioned. The “bottom-up” approach frequently failed, especially within government agencies, due to poor communication and bureaucracy by duty bearers. For instance, an NGO representative reprehended a local social worker in Ghana, also a participant of the FGD, for failing to highlight important matters to the regional office of the social welfare department.

*“Your regional director for instance doesn’t know it is a serious problem, you have not been able to communicate from the district to the regional office...”*

- FGD, District representative, NGO

Ultimately, the majority of the participants stated that 1000 days plus policies often lacked clear objectives. Even when the priorities were stated, they often did not match other stakeholders’ priorities because they either contradicted the stakeholder’s mandate or were perceived as not important. The results from the FGDs suggested that 1000 days plus policies were created with a very top-down process and lacked shared priorities which reduces stakeholders’ ownership leading to insufficient communication between key stakeholders. The majority of the participants emphasized the necessity of defining clear policy goals and priorities. In setting common shared priorities, government representatives warned other members present at the FGD about the role of political interference while identifying the policy’s priority and about the need to address the constantly changing political environment.

*“As a political institution, so it is very difficult for me to talk about [priorities] because as government keeps changing new policies keep coming up. …, every government comes in with his or her own priority*.”

- FGD, District representative, Municipal Assembly

#### 3.1.2. Sustainability

Representatives of the national and sub-national levels, from non-governmental organizations (NGOs) to government agencies, widely agreed that sustainability is a key challenge for the long-term successful implementation of the 1000 days plus nutrition programs and policies. Financial sustainability appears to be an especially major issue. From FGDs and IDIs, it emerged that many NGO programs were not addressing, during their policy planning, the long-term financial sustainability of their projects.

*“We need to begin to think about how we can sustain this project after we pull out*.”

- IDI, District representative, NGO

Unanimously, all participants, including representatives from NGOs and donor agencies, agreed that financial sustainability should be a priority for all the stakeholders involved. Government official representatives in the FGD claimed that they are often expected to sustain the programs after donors and international NGOs pull out. Nevertheless, their budget does not allow them to finance the projects, leading generally to an abrupt end. Moreover, NGOs often would not consult and involve them during the planning phase, but nevertheless expect them to take over the program in a later phase. Most participants indicated that, to avoid this scenario, the sustainability of the 1000 days plus intervention should be addressed at an early stage of the policy and program planning and all key stakeholders should be involved.

*“I don’t want to believe this, pulling out and expecting us sustain them… there should build sustainability in their program so that when they are pulling out, it doesn’t become so harsh.”*

- FGD, District representative, Agric department

#### 3.1.3. Stakeholder Collaboration

Every stakeholder recognized the long-term benefit of a holistic approach to the 1000 days plus nutrition policy framework, but they found it challenging to implement it. Clarity on the division of roles and responsibilities among the different stakeholders is missing. NGOs and government agencies said that duplication of effort is a common challenge that leads to missed opportunities for stakeholder collaboration. Especially in low resource settings, this should be avoided. Even when some of the NGO representatives claimed to have already tackled the duplication of effort, it still emerged as a crucial challenge from the FGDs. As a possible solution, government representatives advocated for an NGO consortium to foster more collaboration during program implementation to curb duplication.

*“...Nutrition is not a one way, it cuts across everywhere, and be it education, health, agric, all aspects of life…we need to target the duty bearers strengthen their inter-sectorial collaboration.”*

- FGD, Local representative, Municipal Assembly

This notwithstanding, it was discovered that there was some level of resistance to initiating possible collaborations. The core reason was mistrust among different stakeholders due to competition for scarce resources at national and sub-national levels (including governmental and nongovernmental implementers). Financial, infrastructural and human resources appeared to be key determining factors when establishing new collaborations. During FGDs and IDIs, it emerged that organizations such as the Ministry of Gender, Children and Social Protection (GCSP), which plays a crucial role in the 1000 days plus nutrition policy, is inadequately resourced and hence lack capacity to lead collaborative efforts. To improve inter-sectorial collaboration, joint funding opportunities should be explored and communication should be promoted during the implementation phase. Since nutrition cuts across many sectors, many respondents recognized the need to have a policy that integrates the multi-level and multi-sectorial approach providing a platform for communication and collaboration.

*“So I see a lot of lack of coordination and Agric is talking the same, but at the district level. There should be an integrated team that is geared towards one goal…we really need to discuss this as a team because when you get to the field..[you need] that kind of coordination and integration.”*

-FGD, Local representative, Ghana healthcare system

### 3.2. Resources

#### 3.2.1. Financial Resources

All stakeholders unanimously agreed that adequate funds are essential for a successful policy implementation process. The majority of government and NGO stakeholders reported their experience of scarce and erratic flow of funds. Government funds, as for donors’ funds, are sometimes inconsistent and irregular. Moreover, within government agencies, the scarce funds have to be prioritized and divided among the different departments, as explained by a municipal assembly representative. Often, the scarce budget does not include the essential costs needed for a successful implementation, such as travelling costs, and this hinders the implementation phase.

*“... Imagine riding a motor bike from here to Naaga with only 5 cedis fuel, I don’t know how you are getting there.”*

- FGD, Local representative, Agric extension officer.

To limit this, as indicated by many participants, there are currently three main ways for the local governments to obtain and acquire financial resources for the 1000 days plus policies and programs: (1) taxes or levies; (2) NGOs and donor agencies; and (3) government funds. Taxes are the major source of funding especially at the municipal level. However, representatives from government agencies stated that many citizens have the attitude of not paying market tolls and basic taxes. NGO and donor agency funds are essential but limited to a time frame and not sustainable in the long term. Government funds contribute the greatest to the 1000 days plus budget. Nonetheless, they are not sufficient to provide an adequate budget for the nutrition department. To resolve this issue, government agencies have been thinking outside of the box and searched for alternative solutions to finance the 1000 days plus policies and programs. The Agriculture department recently adopted a self-finance approach through fee charges for food demonstration and income generating activities through micro-finance projects, especially for women, in collaboration with the Ministry of Gender, Children and Social Protection of Ghana. These have been proven to be relatively successful. Nevertheless, during FGDs, the danger of adding fees to the program was discussed. Program implementers need to be aware that adding a fee may reduce accessibility to the program itself.

*“Our biggest challenge is the erratic flow of funds; funds don’t come, when they are expected, so it slows down the progress of a particular intervention. Sometimes, they don’t even come at all.”*

- IDI, Local representative, Municipal assembly

#### 3.2.2. Human Resources

There was overall agreement during eight FGDs and IDIs that lack of capacity building and staffing are key obstacles. The system in which the healthcare workforce is trained has changed, which according to a GHS participant led to a lower quality of the training and the loss of institutional memory. All participants mentioned that capacity building should be a priority. Investing in sustainable capacity building is needed and possible as the United Nations (UN) representative claimed.

*“We build their capacity and they in turn train those who are going to implement* it.”

- FGD, International representative, UN

*“I think capacity building of the health staff and the volunteer is very critical yeah so that they can give effective counselling to the mothers to be able to care for the children.”*

- IDI, Local representative, Nutrition Department

The majority of respondents from government agencies expressed a need for training on nutrition-related skills but they also mentioned that the government does not have enough funds to sponsor it. Therefore, additional external support was needed. Donor agencies and NGOs are currently investing in building capacity for every player involved such as “health workers, agric extension officers, WASH officers, Social welfare”, as mentioned in one FGD and two IDIs. They train them on soft and nutrition-specific skills related to the 1000 days plus nutrition programs. Training is only a part of the solution to improve capacity building. Many respondents mentioned in four IDIs and FGDs that adequate staffing is also needed. Lack of healthcare professionals is a great challenge.

*“The challenge is staffing, you have only 4 nutrition officers for the whole district.”*

- FGD, District representative, nutritional officer

Lack of motivation is a key issue to get staff engaged, as an NGO representative explained. During an FGD, GES (Ghana Education Service) representative provided the example of the Girls’ Iron-Folate Tablet Supplementation (GIFTS) program. During the implementation of the program, there were instances where local teachers, not wanting to have an additional duty, actively discouraged the children from participating in the GIFTS program, hence hindering its implementation. To aggravate the matter even further, incentives are lacking for overworked staff to remain engaged such as salary reward and trainings. Nevertheless, it appeared that, particularly in government positions, financial incentives are an effective tool in motivating workers, as mentioned in one FGD and two IDIs. As a local GHS representative said, the lack of incentives with an unfitting work mentality and a shortage of human resources is a big challenge.

*“We don’t have a lot of pediatricians in Upper East but I think there is just one so, they needed some support, external support.”*

- IDI, International representative, UN

In this context, volunteers appear to be a key human resource according to all respondents. In three IDIs and one FGD, representative from GHS, GES and other government sectors claimed that lack of volunteerism to support healthcare workers lead them to experience work overload.

#### 3.2.3. Infrastructure

Several stakeholders also mentioned that the infrastructure and equipment were not sufficient to meet the actual policy requirements as stated by government representatives across different departments and NGOs in three IDIs and one FGDs. A GHS representative said that, in the growth and monitoring program, there are not enough infantometers, an instrument for measuring babies’ length. Another local healthcare worker also added that laboratory equipment for the first contact hemoglobin (Hb) measurement is insufficient and healthcare workers have to refer the patients to other facilities capable of measuring Hb levels. This usually results in some clients not able to access the services and hence impacting on their health and the growth and health of the unborn child. Sometimes the supplies are there but not locally available, as mentioned in two IDIs and FGDs. At other times, there are supplies for the moment, but it is unclear if in the future they will be available leading to lack of sustainability of services. Providing physical infrastructure, including services, is currently a key priority at the municipal assembly level.

*“The Infantometers that I even spoke about, they are not enough, it is just a few facilities that are taking such measurements.”*

- FGD, Local representative, GHS

Infrastructure for policy implementation is not only limited to specific equipment and facilities. It extends to organizational structures such as institutions and partnerships. In this regard, several stakeholders expressed the view that the partnership network should be a better integrated team. They recognized that institutions require a platform for different partners to initiate collaboration. Key obstacles to establishing a well-functioning partnership network, as mentioned by multiple representatives, are bureaucracy and challenging logistics, which mostly characterize government institution infrastructure. They delay and prevent institutions and key players from achieving stated goals.

*“Sometimes you want to work with a particular department but the process… bureaucracy sometimes it delays, it delays a lot of processes and you also want to deal with an institution and then you are dealing with director.”*

- FGD, District representative, NGO

#### 3.2.4. Media

Most participants mentioned during FGDs and two IDIs that media have a facilitating role in the policy implementation and the feasibility of the 1000 days plus programs. Market play, drama series, TV shows, radio and songs were identified by NGO, government agencies and civil society representatives as an effective communicating tool. They are currently sporadically used in the implementation of several NGOs and governmental programs. The potential of media for health promotion initiatives has yet to be fully explored, as NGO, GHS and UN representatives mentioned. Nevertheless, it appeared from FGDs that media can reach a diverse and vast target group. Since almost every person is connected to a media tool, it offers the possibility of reaching a bigger target population and continuing to repeat systematically key messages inherent to the first 1000 days plus nutrition policy. Several stakeholders also agreed that media platforms could support even further the communication and shape policy discourse.

*“We are also working with the media to be able to shape policy discourse and also address some of the myths.”*

-IDI, District representative, NGO

*“We also have a project called Communicate for health so as the name their role is to basically to work to support the Ghana, the health promotion unit/department of the mental health Ghana Health Service. They support the government to do nutrition messaging aids.”*

-IDI, International representative, donor agencies

#### 3.2.5. Monitoring System

Several stakeholders mentioned during five IDIs and FGDs that the monitoring and evaluation system is still being developed and it does not yet meet their needs. The lack of effective surveillance does not allow a proper assessment of the policy impact and its implementation. Government representatives highlighted that crucial data on nutritional status, used to guide implementation, evaluate policy and obtain funds, are missing. The current nutrition surveillance does not allow effective follow-up of the implementation process and identification of the best-practices because it lacks an effective monitoring system to collect crucial data. An NGO representative said that GIFTS program provided data on stunting but data on the nutritional status of children are still lacking. In most cases, to advocate for a matter to be placed on the political agenda, evidence is needed. As all stakeholders pointed out, the lack of data and monitoring system does not allow determination of the core problems, identifying the most effective solutions and assessment of the effectiveness of the current implemented programs.

*“GIFTS has come to give us a lot of data on stunting but I was thinking, we still lack data on the nutritional status of children...”*

-FGD, District representative, NGO representative

### 3.3. Leadership and Stakeholder Engagement

Most respondents stated that leadership of duty bearers, especially supporting local and regional leaders (or “champions”), improves the implementation processes. Several stakeholders reported that they had empowered “champions” as local leaders to become implementers themselves. Champions are usually respected individuals within the community, and they have the ability to influence the other community members by advocating and promoting positive change on behalf of the implementers. Nevertheless, due to lack of motivation and access to remote areas, among other reasons, they are difficult to engage. To increase the number of local “champions”, government representatives argued that stakeholders and the target population should be meaningfully involved in the early phase of policy making processes at local and regional levels. This has the potential to reduce policy implementation gaps caused by lack of effective leadership.

*“The other component was on improving leadership, governance, coordination of new born care programming. Because without that you will not be able to have the results that you need.”*

-IDI, National representative, UN agency

In four IDIs and FGDs, all stakeholders mentioned that sometimes only donors are setting the agenda, excluding beneficiaries and other stakeholder from the discussion. Several stakeholders, especially from government agencies, complained that those donors with financial resources (notably international donors) have more decision power and they often decide who to engage in the discussion by marginalizing other key actors. This demonstrates one of the many missed opportunities to strengthen stakeholder engagement in the 1000 days plus framework.

*“When they come, they really don’t listen to how you want it, they want to do it their way because they want results, once they get the results they are gone then you have a problem.”*

-FGD, Local representative, GHS

Participants perceived that there was need to create a space to promote meaningful stakeholder engagement among and within sectors. There are initiatives at the municipal assembly level that have achieved successfully a more inclusive stakeholder engagement approach especially with various community members as claimed by municipal assembly representative Many stakeholders recognized a stakeholder analysis as the most appropriate starting point. The different stakeholders at different entry points have to all come together to be one, as mentioned in eight IDIs and FGDs.

*“One of the strategies we could have adopted is … a stakeholder analysis and who to engage and where, we will realize that there are certain areas that we need to prioritize so based on the skill set that we have.”*

-FGD, Regional representative, NGO

### 3.4. Implementation Guidance and Ongoing Communication

Most stakeholders, especially government representatives, perceive a lack of clear guidance regarding expectations and distribution of responsibilities during the 1000 days plus policy implementation. They claim that national policies are not adequately communicated to local key players. Government and NGO representatives stated that sometimes their partners, especially donor agencies, showed a “top-down” approach with poor implementation planning. Often NGOs, during their policy implementation, are not addressing the priorities and needs of the local level key actors which lead to a lack of communication of responsibilities, expectations and retributions. For example, during FGDs, lack of communication regarding job expectations appears to be the leading cause, among other factors, behind shortage of volunteers. Local government and NGO representatives should be more transparent in the ongoing communication and collaboration with the key actors.

*“They should just be specific, this is what they are going to do, there is nothing they are going to offer you, it is for the benefit of the community… but they shouldn’t keep quiet as if at the end of it there is nothing, you can see that surprises.”*

-FGD, Local representative, volunteer

The majority of stakeholders agreed that there is a gap between the national and the local level, often due to a lack of guidance and ongoing communication. It appears that there is a mismatch between the national policy goals and the actual attainability of the proposed objectives in relation to the local context due to a lack of effective communication.

*“I also say that as far as I’m concerned there’s always a gap between National and district level because they have beautiful policies well thought through then it ends there.”*

-IDI, International representative, donor agencies

### 3.5. Organizational Culture

Several respondents, from governmental and non-governmental organizations, said that differences in the organizational culture among the stakeholders negatively affect the implementation of 1000 days plus initiatives. The majority of the stakeholders prioritize individual interests. Acknowledging and addressing those individual motives and differences, often related to a “what is in it for me?” mindset, can lead to a successful implementation. These differences also extend to how different organizations work and are structured. In most cases, government agencies are looking at vertical growth while donor agencies and NGOs are looking at the horizontal growth. The duration of policy greatly varies among stakeholders. For example, UN organization work is structured based on a five-year working plan, whereas political organizations, in comparison, have more variability and less predictability in their work since local governments keep coming up with new policies for implementation. In five IDIs and FGDs, many stakeholders identify the need for alignment of the different working plans to avoid gaps during the implementation phase. If not, it can happen that reaching the target goal by one partner can lead to the interruption of the program, hence hindering the other partner from achieving their target goal. This was the case for the “plumpy nuts” program, as mentioned during three IDIs and FGDs. In general, the government representatives claimed that some donors’ practices have negatively affected the working attitudes of their staff, as the practice of providing financial reward to volunteers is not a common method used in government agencies. They claimed during FGDs that this practice discouraged repeated volunteerism in the government sector.

*“The commitment is not there because there is nothing nobody is really ready to go the extra mile, I think we should change our attitude towards work.”*

-FGD, Local representative, GHS

The organizational culture also has an effect on how programs are financed. During the FGDs, it was mentioned that, while collaborating with donor agencies, it is necessary to be aware of the type of financing system they utilize, in order to secure enough liquidity to ensure the continuation of the program.

*“You are able to differentiate donor programs and the government programs so that you would have more expectations for the donor ones and then less expectations for the government ones.”*

-FGD, Local representative, volunteer

### 3.6. Accountability and Governance

Accountability provides the basis for creating good governance [31]. Overall, the stakeholders agreed that lack of accountability, and consequentially reduced governance, are key barriers to policy implementation. Nevertheless, the majority of stakeholders perceived that government agencies in particular do not enforce accountability especially when it comes to the distribution of responsibilities. Key stakeholders are not aware of the different duty bearers’ responsibilities and are therefore unable to enforce eventual agreements and make duty bearers accountable for their mandate’s areas. The matter is aggravated when there are more organizations, institutions and partnership agreements involved. In two IDIs and FGDs, the Nurturing Care Framework for Early Childhood Development (NCFECH) was mentioned as a good practice to hold duty bearers accountable. Unfortunately, this framework is not specific to the first 1000 days plus nutrition policies and programs, but it is limited to a circumscribed policy area. Since the 1000 days plus nutrition policy area includes broader objectives and multiple implementing actors within and beyond the health sector than does the NCFECH, it is more challenging to execute a stakeholder analysis and hold duty bearers accountable. Nevertheless, more accountability in the 1000 days plus initiatives could have many beneficial effects. According to the FGDs and IDIs, it has the potential to increase the ownership, expectations and commitment of community members by forming new local leaders. In turn, this will support local implementation and benefit the long-term sustainability of the 1000 days plus policy framework. For these reasons, government and non-governmental agencies have tried to focus more on creating ownership in their programs. Nevertheless, as an NGO representative mentioned, ownership is still lacking.

*“… If we are able to build the capacity of all the stakeholders around that [connect the respective mandates], so that everybody is seeing this thing in their respective mandates, what are the opportunities within our respective work which promote this 1000 days...”*

-FGD, District representative, NGO

### 3.7. Coverage

Overall, the stakeholders concluded that the coverage of the 1000 days plus policies and programs is not optimal. The implementation’s needs differ from one region to another. These regional differences require flexibility in the provision and planning of the services that is not available in the current setting. The most vulnerable and unreachable populations, such as in rural areas or orphaned children, are not effectively targeted by the programs. The stakeholders identified three main barriers to improving coverage. These were related to availability, accessibility and acceptability:

Several stakeholders identified scarce availability of resources compared to the size of the target population, again highlighting regional differences. In particular, sourcing and provision of nutritious food and micronutrient security was identified as a major challenge. The government is encouraging the private sector to meet national needs and the communities to consume nutritious local food as a representative of the Agriculture Department illustrated during IDIs and FGDs.

*“Food that is locally available for the community... linking farmers to the potential of it and encourage them to pick it up.”*

-IDI, District representative, Agric department

Accessibility to the service is limited and unequally distributed throughout Ghana, as NGO and Government representatives mentioned. The stakeholders mentioned temporal, financial and spatial barriers. In rural areas, health facilities are not close to the target population and implementing programs is a major issue. The stakeholders recognized community-based volunteers and champions as a potential solution to increasing accessibility. However, in the eventuality that nutrition services are available, they are sometimes not affordable, as mentioned during FGDs. A local government representative provided the example of the food demonstration where the participatory service fee can be a financial barrier, because it renders the services unaffordable to poor and vulnerable households that should be its target. Government programs are trying to address these barriers.

*“[Through Leap 1000 program] they are able to purchase food from the market, probably balanced diet will improve their nutrition and then, as part of the package beneficiaries get free health insurance...”*

-FGD, Local representative, Municipal Assembly

Several stakeholders mentioned that, even when resources are available and accessible, the population does not make use of them because of culture, beliefs, religion and gender norms. Acceptability of the services is therefore a challenge. Taboos and myths negatively affect the implementation coverage. The GIFTS program was particularly affected, as GHS representatives mentioned. The misconception that the iron and folic supplementation, implemented as part of the GIFTS program, were family planning drugs lead to a decrease in coverage. Lack of knowledge and community sensitization programs are at the origin of these misconceptions. In five IDI and FGDs, the majority of respondents recognized that gender norms on the role of women’s and men’s involvement also negatively affect the coverage. This is because gender biases lead to decreased male participation. Husbands, together with grandmothers, are key actors in the family power structure and influence the nutritional intake of mothers. When power holders and decision makers within the family structure are not targeted by the nutrition programs, the policies are often not successfully implemented, as mentioned in six IDIs and FGD. Engaging these power structures around creating an enabling environment for mothers is a key priority. In the policy planning, local government and NGO respondents in FGD said that the national government did not take sufficiently into consideration the cultural differences between regions.

*“One of our challenges is that, most of the policies that come up, they don’t really fit into our cultural norms, because mostly whatever that is best practices that can happen in Accra is different from what will happen in Navrongo.”*

-FGD, District representative, NGO

## 4. Discussion

This qualitative study critically analyzed perceptions of key stakeholders regarding the implementation process of nutrition policies within the 1000 days plus policy landscape in Ghana. Stakeholders identified critical areas related to policy development and implementation as mentioned below.

### 4.1. Single 1000 Days Plus Nutrition Policy

Ghana is in need of a comprehensive 1000 days plus nutrition policy to work towards a shared vision in improving early life nutrition and sustainable outcomes. This is in line with other studies that showed the importance of having a single policy, with clearly formulated and attainable objectives drafted and shared among all involved stakeholders [5,21,30,31,32,33,34]. Ghana currently has different individual policies and interventions that jointly contribute to a broader policy framework for nutrition and which include, but are not limited to, the 1000 days plus nutrition period [3,5,19,21]. Even though many stakeholders shared the importance of the 1000 days plus concept, they never translated this into a single overarching policy. Many 1000 days plus nutrition policies and program originated from other sectors than health which weakened the leadership from the GHS and in particular of the Nutrition Department. This led to fragmentation and duplication of the 1000 days plus nutrition policies and program, with each of these having their individual sector mandates, priorities and functions. This was partially caused by a lack of platforms to engage and ensure intersectoral collaboration and coordination. Stakeholders identified the Nutrition Department as a poor leader incapable of building, guiding and creating a 1000 days plus nutrition vision. This study also reinforced previous findings that identified the Nutrition Department as a low-ranking agency within the GHS power structure, receiving limited and inadequate resource allocation [5,21,30,31].

The study participants did not indicate methods that could be helpful in formulating the 1000 days plus nutrition objectives. Ghana could however use the specific, measurable, achievable, relevant and time-bound (SMART) framework to formulate policy objectives as other governments have done [32,33,34,35]. Ghana could plan a single policy based on investigating which current 1000 days plus policies and programs are successful and scaling these up. To achieve this, as identified in this study and in line with the policy planning literature, a system of monitoring and evaluation that provides data on availability of resources, a detailed inventory on context-specific preconditions and data on the nutritional status of the entire Ghana in population, is needed [17,21,36,37].

### 4.2. Policy Implementation Strategy

Even in the absence of a comprehensive policy, current nutrition policies and programs in Ghana still need to include an effective policy implementation strategy [24,25,29,38,39,40,41,42]. This study found that implementation was hampered by a poor division of responsibilities and mandates among the key stakeholders at all implementation levels. This, coupled with a lack of an effective monitoring and surveillance system, did not permit measurement regarding policy impact and identification of best-practices in Ghana. Moreover, in line with evidence on HIV/AIDS control and donor influences, the availability of donor funds for nutrition activities provoked competition and hampered collaboration among implementers [43,44]. In addition, some organizational culture differences between NGOs/donors and government agencies negatively impacted the implementation process [45]. The study reinforced previous findings on the benefit of engaging implementers and donors in the early phase of policy planning to ensure harmonization and programs alignment within country priorities [36,44,45]. It also emerged from this study that the shortage of resources was hindering the implementation process itself. In particular, as unanimously mentioned by all stakeholders, capacity building, system monitoring and funding stability would be key resources needed for successful implementation of the 1000 days plus policies and programs in the future. The findings of this study are largely consistent with themes identified by a wide range of health policy implementation studies [5,17,28,29,30,31,37,38,39,40,41,42,43,44,45,46,47,48].

A strategic action plan should have been developed to support policy makers to implement their visions, increase efficiency and guide the implementation of the 1000 days plus policy and programs [23,25,29]. Apart from a clear vision and mission, there is a need to define the critical areas for action. Considering the shortage of resources, this study identified seven core themes (planning policy implementation; organizational culture; implementation and ongoing communication; leadership and stakeholder engagement; accountability and governance; resources; and coverage) and ten sub-categories that should have been prioritized. These themes could be seen as the critical success factors or critical action areas, vital to 1000 days nutrition policy and programs successful implementation.

## 5. Strengths and Limitations

Our study has many strengths which we would like to highlight. This study is a unique and novel research on its own since it is among the first studies to investigate the policy nutrition landscape around the first 1000 days plus period. In particular, it focuses on the implementation process of the 1000 days plus nutrition programs and policies, a topic that in the last year has emerged as crucial but nevertheless still under researched. Our study closely relates to bridging the “knowledge–do“ gap and to improve maternal and child health in low- and middle-income countries.

We also wish to acknowledge some of the limitations of our study. Firstly, not all key stakeholders, such as representatives of academia and the media, were interviewed. We believe inclusion of a wider stakeholder representation would have offered a better insight of the implementation process. Although the implementation processes for policies in the GHS is similar in the entire country, the implementation challenges may differ and hence our results may not be fully generalizable. This notwithstanding, we believe the study thus offer important insights on the subject matter and also indicates an area for future research to cover other settings in the country.

## 6. Recommendations and Future Research

While this qualitative study has started to build a general understanding of the main barriers and facilitators to the implementation of the nutrition 1000 days plus activities in Ghana, several knowledge gaps remain. Key recommendations for policymakers generated from the findings of this study are as follows:

There is an urgent need to conduct a stakeholder analysis that investigates the individual mandates, skills and resources to promote stakeholder collaboration, to prevent duplication of effort and to clearly define the mandates of the duty bearers at all implementation levels:

1. A stakeholder analysis of all involved 1000 days plus key actors.
2. An inventory of the first 1000 days plus nutrition programs and policies that are currently being implemented in Ghana should be performed.
3. A resource analysis should be conducted with the aim of critically evaluating the current and future resource capacity.
4. A single 1000 days plus nutrition policy, based on SMART nutrition indicators, should be formulated, shared and adopted by all the relevant stakeholders.
5. All key actors should be meaningfully involved from the early phases of policy planning.
6. A surveillance and monitoring system should be established to track and guide the implementation phase of current policies and programs.
7. New indicators that better reflect the impact of policies on nutritional status of mother and children should be identified.
8. Research should further investigate the origin of the cultural barriers and regional differences that hinder policy implementation.
9. A communication platform exclusive to the 1000 days nutrition policies and programs should be established.

## 7. Conclusions

To eradicate malnutrition by 2030, actions to improve early-life nutrition are essential. Despite recent progress in nutrition indicators in Ghana, results have not always been achieved due to a lack of a single specific 1000 days plus policy and implementation plan. In this study, stakeholders in maternal and child nutrition identified several obstacles to policy implementation around the first 1000 days plus period. Policy planning that tackles the contextual barriers identified in this study, coupled with an effective monitoring system to measure its impact, will assist in identifying best-practices and promoting a successful 1000 days plus policy implementation. This is of even more importance in the light of the double burden of malnutrition in Ghana and for the realization of policy goals for double-duty actions to address malnutrition in all its forms.

## Figures and Tables

**Table 1:** Stakholders level of operation and role in the policy process.

Stakeholder	Level of Operation	Role in Policy Process
**Government Agencies**		
Ministry of health	National	Policy generation
Ghana Health Service—Public Health	National Regional Municipal District	Policy implementation, monitoring and evaluation
Ghana Health Service—Nutrition	Regional Municipal District	Policy implementation, monitoring and evaluation
Ministry of Education	Municipal District	Policy implementation, monitoring and evaluation
Ministry of Agriculture	Regional Municipal District	Policy implementation, monitoring and evaluation
Ministry of Gender, Children and Social protection	Municipal District	Policy implementation and monitoring
Local Government Authority	Municipal District	
Community based organizations	Community	Ensure community participation in implemented policies
Community health volunteers	Community	Ensure community participation in implemented policies
**Donor agencies**		
UN agencies	National	Policy generation, implementation and monitoring
Other non-governmental organizations	Regional Municipal District	Policy generation, implementation and monitoring

## Data Availability

The data presented in this study are available on request from the senior authors on this paper. The data are not publicly available due to strict confidentiality issues.

## INPreP Study Group Co-Investigators and Researchers of the INPreP Study Group Include

Karen Hofman, Hermann Sorgho, Mary Barker, Caroline Fall, Cornelius Debpuur, Romuald Boua, Agnes Erzse, Sarah Kehoe, Daniella Watson and Polly Hardy-Johnson.
